# Electricity and Its Applications in Dentistry

**Published:** 1896-11

**Authors:** William E. Clark, Philip W. Davis


### ELECTRICITY AND ITS APPLICATIONS IN DENTISTRY.[1]

BY WILLIAM E. CLARK, S.B., AND PHILIP W. DAVIS, A.B., S.B.

After considering what ought to be the best way to treat of the nature of electricity and of its applications in dentistry, Mr. Clark and I decided that our aim should be to give to those of you who do not thoroughly understand the nature of electricity, but who intend making use of such of its applications as are at your disposal, the power to make an intelligent investigation for yourselves, and to enable you to come to a decision concerning what electrical apparatus will best serve your individual purposes and how to select it. At the same time we shall not be sorry if we succeed in arousing an interest in the use of this agent among those who think that electricity is still in its infancy. In dealing with this subject it is important not to neglect the theoretical side for the practical, since a correct conception of the former is essential to a thorough understanding of the latter. Nor do we think it well to omit a brief mention of the historical side of the subject, if only for completeness' sake. In trying to make ourselves clear to those who have not made a special study of this question, we may at times seem elementary to such among you as have, and we must beg your indulgence.

Some two thousand years ago the Greeks were in the habit of buying bits of amber which the Phœnician traders brought from the shores of the Baltic. They wore them as amulets, because they

---

[1] Read by Mr. Philip W. Davis, of Cambridge, Mass., before the American Academy of Dental Science, at Young's Hotel, April 1, 1896.

had noticed that when rubbed against cloth a piece of amber would attract small pieces of straw and other objects, and they regarded this phenomenon as an evidence of a mysterious sort of life which they called the "soul of amber." Now in the course of ages this little bit of knowledge was handed down, and it was also gradually discovered that other substances possessed the same power. At last this property of attraction became known as ἤλεκτρον, which is the Greek word for amber, and from this is derived our word electricity.

The phenomena of frictional electricity were studied scientifically as far back as the sixteenth century by one Dr. Gilbert, body physician to Queen Elizabeth; but little was accomplished until the discovery of the Leyden jar by Dean von Kleist in 1748, who found that electricity could be imprisoned or accumulated in a glass jar coated inside and out with a conducting material.

That brilliant quack Mesmer, who turned Paris topsy-turvy, was probably the first man to use electricity on the human body. But the inventors who laid the foundations of modern electrical science were Galvani and Volta, who invented the electric battery in the middle of the eighteenth century; Ohm and Ampère, physicists who discovered the laws of flow of current; Oersted and Faraday, who discovered the laws of induction. Since then there have been many investigators of wonderful ability, but it is the names that we have mentioned that you will meet with in the course of your inquiries in the electrical field.

The problems concerning the nature of electricity and the laws governing its actions are as well known to physicists as the laws governing most other phenomena of nature. This may seem a little remarkable at first thought, but it will not seem so strange when it is remembered that there have been many great workers in this field, and also that many of the wonderful strides which have been made in the other sciences are of quite recent date.

Electricity is a form of energy, is subject to the laws governing all other forms of energy, and can be readily converted into any one of them. There are two characteristics which determine completely the amount of energy present in any circuit. These are intensity and quantity, and they have been named after two celebrated physicists, the unit of quantity being called the ampère, and the unit of intensity the volt. It is this method of naming electrical characteristics, born of a desire to honor the great ones of the science, which has loaded it with many peculiar words so confusing and mysterious to the uninitiated.

In order that a flow of electricity should occur between two bodies one of them must be at a higher potential than the other. To explain this let us make use of an example. Imagine two long pipes closed at one end, while at the other is some mechanical device which shall force air into one, raising the pressure a given amount, while sucking it out of the other, reducing the pressure an equal amount. Then if anywhere along the line a small tube is inserted connecting the two mains, a flow of air will immediately occur from one to the other, depending in its quantity on the friction due to the small tube. This, gentlemen, is analogous to a complete electrical circuit. For pipes substitute wires and for the pump substitute a dynamo. The friction of air in the tube has its electrical counterpart in a property which a wire possesses of offering more or less resistance to the passage of a current, according to the cross section of the wire and the material of which it is composed. The unit of resistance has been called an ohm.

There is a simple law which governs the relations of these three quantities to each other, and it is this: that the pressure in volts, divided by the resistance to flow in ohms, is equal to the quantity in ampères. It has been stated that the pressure and quantity determine the amount of energy in a circuit. The pressure in volts, multiplied by the quantity in ampères, gives the energy in watts, a term derived from the name of an inventor who did more than any other to further the possibility of transforming one form of energy into another. I mean the inventor of the steam engine.

And now a word about the dynamo and its construction. It depends for its principle on the fact discovered by Faraday, that every wire carrying an electric current is surrounded by a magnetic whirl, and that whenever one of these whirls cuts across another wire a current is at once induced in it. When a long wire is coiled together into a spiral the magnetism is concentrated through the coil. Soft iron has the property that when introduced into this spiral it increases the magnetism present enormously. The iron is extended beyond the coil at both ends and is bent into a U-shaped form, the ends being concaved so that a cylindrical body may revolve between them. In the space between these poles, as they are called, coils of wire are revolved. As they cut the magnetic field extending from pole to pole an electric pressure, tending to produce an electric current, is generated in these coils, and is delivered to the mains where it is ready to set up a flow of current through any device connected with them. The small current necessary to magnetize the field coil is also derived from the mains.

Thus it is very simple to outline how a coil of wire, revolving in a magnetic field, has induced in it an electrical pressure tending to set up a current, but you must not ask the electrical engineer why these conditions produce the electric current any more than you must ask the astronomer why the earth attracts the sun, or the physicist why certain ether vibrations produce light.

The electric motor is much the same apparatus as the dynamo except that its armature, the revolving part, absorbs electrical energy in order to revolve, while the dynamo's armature is revolved by mechanical energy in order that it may give out electrical energy. A small amount of electricity passes through the field coils of a motor, making one pole of the horseshoe a north or positive pole, and the other a south or negative pole. Most of the current, however, passes through the armature, where north and south poles are also established, and these poles being attracted by the horseshoe or field magnets, the armature revolves. There is a sliding electrical contact made by brushes rubbing on a commutator, and by this device the current is conveyed successively through the different coils of the armature in such a manner that its poles are continuously changing with respect to a given point on the armature, but are fixed with respect to the pole pieces, being continuously attracted towards them but never getting there.

Electric currents have many different characteristics. They are of high or low voltage, of constant quantity or of constant pressure, direct or alternating. It would take too much time to explain all these different details, and so only the more important points can be brought out, and those briefly. A direct or continuous current of constant pressure passes through the circuit in the same direction all the time. As it is generally desirable to raise the pressure to one or more thousand volts, when transmitting energy more than a couple of miles, the direct current is often not the most economical one to use, because its voltage cannot be raised or lowered conveniently except by expensive moving machinery. The necessity for using high pressure proceeds from the fact that the loss in transmission is equal to the resistance of the line multiplied by the square of the current, and it is therefore desirable to have the current as small as possible. To do this, and keep the energy in the circuit unchanged, the voltage or pressure must be correspondingly increased, as you will readily see if you remember that the energy in watts equals the current multiplied by the pressure.

The alternating current is one that flows through the circuit first in one direction and then in the other. At one instant there is absolutely no current in the wire, then it rises gradually to a maximum, then falls to zero, begins to flow in the opposite direction rising to a maximum, and falls again to zero. This complete operation is ordinarily repeated about one hundred and twenty-five times each second. The alternating current is usually generated at a very high voltage, transmitted to the place where it is to be utilized, and then transformed down to a safe voltage by means of a stationary transformer. And thus for the past few years alternating currents have been employed quite largely for transmitting energy over areas of scattered population and over long distances. We hope it is now clear why sometimes the direct and sometimes the alternating current is found in various towns and cities.

Hoping that these explanatory and theoretical considerations may have proved helpful, we now pass to what concerns you most, —the applications.

As far back as 1840 it was an evident fact to many scientists that electricity could be used to great advantage for producing light, if only some way could be found for subdividing the current, and many were the unsuccessful attempts made to do it. On October 11, 1878, there was a panic in gas shares on the London Stock Exchange, caused by the announcement that Edison had subdivided the electric light. This was only a false alarm, and it was not until after 1880 that electric lighting became a commercial fact. It was not until then that electricity in dentistry became practicable. There were primary batteries strong enough to do the work, to be sure, but where was the dentist who could afford the time, to say nothing of the expense, required to make such a device operative. Storage batteries were not yet perfected. These outfits were imperfect electrically and mechanically; the battery was a nightmare to the average man, and the motor was a cheap inefficient and unsatisfactory affair.

The advent of the incandescent lamp into the dentist's office brought with it another benefit besides the superiority and convenience of the light. It solved the problem of supply, by placing the current on tap, so to speak, for any and every purpose; and a temptation to improve his methods was put in the way of the dentist. With sufficient knowledge and proper apparatus there are very few processes connected with dentistry which may not be benefited by the use of electricity, and the profession is realizing this fact more and more.

Now a word concerning the commercial currents that the dentist is likely to meet with. And at the outset we wish to correct a wrong impression, that seems to be in the minds of many people, concerning the words "Edison's current." There is no such thing as an Edison current. It is much more proper to say simply an electric current. There is, however, an Edison system of generating and distributing electricity, and this system is so widely used that dentists have come to think of the system as the thing. There are also others in use. The Edison system uses a direct current at one hundred and ten volts constant pressure, and is mostly applied to incandescent lighting. A distinction is made between high and low pressure currents. Low voltage varies from fifty to one hundred and ten, and is used for house service, while the high voltage varies from three hundred to five thousand, and even more, and it should be remembered that this is dangerous to life and property. If high voltage is ever used it should be only under the supervision and advice of a competent engineer.

There are two classes of electric dental apparatus,—that designed for the low voltage current of fifty or one hundred and ten volts, and that designed for the much lower voltage of four or six volts, and so the expression "low voltage apparatus," when applied to dentistry, sometimes means four or six volts, and should not be confused with the term as used commercially. The direct current is usually delivered at one hundred and ten volts, while the alternating is usually at fifty-two volts pressure. For office lighting either current is equally good. So long as a given amount of energy is absorbed in the lamp it matters not whether the current is steady or alternating in direction. The rise and fall of the alternating current is so rapid, as you will remember, that no change in temperature of the filament has time to take place.

One of the most beautiful applications of electricity in dentistry is the use of the various forms of miniature lamps varying in size from a fraction to several candle-power. It is sometimes absolutely impossible to tell whether or not a tooth is perfect simply from its appearance under normal conditions. By placing a small lamp inside the mouth, however, the perfect teeth appear evenly translucent, while fillings, cavities, and dead teeth are clearly pointed out, as there is a marked difference in their appearance. This is well illustrated by the following case. A young girl had suffered for five years from a sore or fistula under the chin. At different times two surgical operations were performed, each of which seemed successful at first, but each proved only a temporary remedy. Her teeth had been examined twice by competent dentists who pronounced them sound. She finally came under the notice of a dentist who was not satisfied with an examination made only by the aid of daylight. He made use of a mouth-lamp, and on darkening the room, was able to see that a tooth was opaque and quite dead. He drilled into it, cleaned it out, treated it, filled it, and another slight operation being performed, a permanent cure was effected.

Mouth-lamps are often operated by primary cells, which, however, is not a very satisfactory method. But they are very successfully operated by storage-cells. A very low voltage commercial current would be the ideal thing. This, however, is not available. Very often a current of about one ampère is passed through a thirty-two candle-power, one-hundred-and-ten-volt lamp, and then through a small mouth-lamp. This does the work, but cannot be very highly recommended. It is in fact sometimes emphatically to be condemned. The energy to produce thirty-two candle-power of light is used when only one or two candle-power is required. This means a vast percentage of waste, although the actual amount is really small, as the time is usually short. When one needs a mouth-lamp at all, one usually needs it badly; so that the expense of using considerable energy for a short time is trifling as compared with the value of the results. But this inefficiency is only a minor fault. The chief objection is in bringing a lamp connected with a one-hundred-and-ten-volt system and capable of conducting considerable current so near to the mouth.

It would be an improvement if the resistance were in two parts, half being placed in each of the leads which go to the small lamp. Wires of opposite polarity should also be kept as far apart as possible. If every precaution is taken, if the best of materials are used, and if one has a thorough understanding of the principles involved, then this system may be recommended This may be a good place to state that the physiological effect of electricity passing through the body is due entirely to the strength of current, and not in the least to voltage, except as increased voltage means increased current. Thousands of volts may be taken if the resistance in circuit is large enough to keep the current within safe limits. A much smaller strength of current than is generally supposed will produce deleterious effects when passed through the human body.

Those dentists who are supplied with the alternating current have a considerable advantage over the users of the direct current in the matter of lighting small candle-power, low voltage lamps, and indeed in running almost all low voltage apparatus. This fact is not generally very well understood by dentists, and very few have made use of it as yet. The transformation of the alternating current from fifty-two or one hundred and ten volts down to four or six for all kinds of mouth-lamps, for hot-air syringes, for cauteries, for root-canal dryers, etc., is safer, more convenient, more economical, and more scientific than methods used with the direct current, where the voltage is reduced by office lamps, or other resistance.

After once getting the commercial current into the office, and having installed the electric light for illuminating his office, the dentist is ready to take advantage of the most useful of all electric dental apparatus, the motor-driven engine. A good engine should be simple, noiseless, easily cared for, flexible, and not too bulky. The moving parts should be reduced to a minimum; bearings should be self-oiling, or else few and easily accessible; the armature or revolving part must be well balanced, and the whole should be so mounted as to minimize any vibrations. If it is fastened to the woodwork felt might be inserted between the frame and the wood, and the bolts could be supplied with rubber washers. The motor should have a half dozen different speeds, although ordinarily, perhaps, but three or four will be required. The usual method of changing the speed is by means of a rheostat, and this should be controlled by the foot of the operator, leaving both hands free at all times. The motor should be reversible, and it should have some kind of a brake, a very ingenious method being to cut the supply off from the armature, at the same time short-circuiting it on itself, thus converting the machine into a dynamo. The rush of current which is immediately generated stops it instantly. The machines on the market which best meet these requirements make use of the direct current. Those who have only the alternating current are under a disadvantage, in that the alternating current motors on the market are rather inferior to the direct current motors when used for driving electric dental engines. This is only to be expected when it is remembered how comparatively short a time the alternating current motor has been in practical use. There are a number of very good constant speed alternating current motors that revolve always in one direction, but, as has been pointed out, the dental engine requires variable speed and the reversibility of the direction of rotation.

Perhaps the chief advantages in the use of the electric engine are that it enables the operator to work more firmly, conveniently, and rapidly, not having to pump his engine by foot-power, and that it enables him to do a longer day's work with less fatigue. There are several minor advantages, but they need not be mentioned, as those already named would seem to be sufficiently great in themselves. The electric engine is found to be such a benefit, luxury, and sometimes almost a necessity to a dentist having a large practice, that, although best supplied from the commercial current, it often proves worth while to go to the bother of installing batteries when no other means are available. It is not uncommon for a man who uses an electric dental engine to say that he would not part with his apparatus for comparatively large sums of money if he could not replace it.

Batteries may be divided into two classes: primary and secondary. For the dentist's purpose the distinction between them is that the energy furnished by a primary cell can be drawn upon only at a very slow rate, as it will furnish only a very weak current. The storage cell, which, as its name indicates, must first be charged by the action of a current from an outside source, is of such a nature that it can give up its energy as rapidly as the operator needs it. The reason for this phenomenon is largely due to the high internal resistance of the primary cell, which cuts down the circuit, while the low internal resistance of the storage cell allows the current to depend for its strength upon the resistance of the outside circuit. Primary cells are sometimes classified under open or closed circuit cells, a closed circuit cell being one which, to be kept in operating condition, must be allowed to pass a current on closed circuit continuously. Such cells usually have two liquids separated from each other, which tend to mix as soon as the current stops flowing. An open circuit cell can be used to furnish a current for only a short time, and must be given long intervals on open circuit, during which it recovers its strength. These qualities, added to the unpleasantness of creeping salts and the time consumed in caring for primary cells, render them very objectionable for dentists' use.

From time to time there appear upon the market primary cells purporting to be the results of years of study, which are guaranteed to put in the hands of the dentist a store of energy which will work all his apparatus quite satisfactorily. These cells are almost all variations of the chromic acid cell, and cannot be classed under either the open or closed circuit type. They will furnish a fairly strong current it is true, it being possible to run a lamp or a small motor with five or six good-sized cells; but they will consume considerable time and care and a good deal of money in zinc and chemicals, and they have but a short life.

If a battery must be used let it consist of storage cells, and have them charged by some one who makes a business of doing such work, or, still better, install a set of closed circuit gravity cells which are to be kept connected with the storage cells night and day. The energy delivered by the gravity battery is accumulated by the storage battery, and is thus made available. To insure success, however, the gravity cells must be set up properly in the first place, cleanliness must be observed afterwards, and the water must be replaced as fast as it evaporates. Many people have had sad experience in handling batteries, and will advise others to have nothing to do with them. We are only too glad to admit the excellence of this advice when the proper commercial current is available, or if the batteries are likely to receive little or no care. This system calls for about three storage cells, of fifty ampère hours capacity each, and from nine to eighteen gravity cells. If the cells are to be sent out to be charged there should be two sets. These cells should be of one hundred and twenty-five ampère hours capacity. We will repeat once more that a small amount of attention at frequent intervals, an understanding of the nature of the particular cell used, and perfect cleanliness are the chief points to be considered in caring for a battery. We may appear to have given this subject undue space, but we wish to clear the matter up and put it in its true light.

Electrical energy may be used to great advantage for heating water for the water-syringe, and for sterilization, for heating air for the air-syringe, for producing an intense heat for fusing porcelain, and for annealing gold and softening gutta-percha. The electric gold annealer takes the place of the alcohol lamp or bunsen burner, and has many advantages over these for annealing gold. In it a continuous heat may be obtained of fixed intensity. If a rheostat is used in connection with it a number of different degrees of heat may be produced, and so the best temperature for any special gold may be obtained. A much lower temperature is required for gutta-percha than for gold, and it may be set on a tile or soapstone slab which is placed on the annealer. The gold may be put on the annealer before the operation of filling the tooth is begun, and, as the necessary heat required to anneal the gold is not enough to burn it, it may be kept heated continuously while the filling is going on. The heat not being great enough to withdraw the temper from the plugger-point, the gold may be picked up directly without transferring from pliers to plugger. In this way the work goes on rapidly, and much time is saved.

The use of the alcohol lamp has been called a nuisance, for it is dangerous and it is expensive. A bit of carbon caught up from the wick might render a gold filling worthless. It is impossible to anneal gold evenly by holding it with a pair of pliers and passing it through a flame. The edges may be burned before the part touching the steel is fairly warm, for the steel conducts the heat away from the gold very rapidly. With the use of the annealer, however, the gold is not only heated to the proper degree, but the annealing is absolutely uniform.

In closing it might be well to summarize the various pieces of apparatus. The pieces that have a large use at present are the engine, the motor for running the laboratory lathe, the office lamps, the mouth-lamps, the electric fan, the hot-air syringe, the root-dryer, the cautery, the hot-water heater, the electric furnace or oven, the gold annealer, the soldering iron, and apparatus for producing local anæsthesia.

We have outlined a good many applications of electricity in dentistry, and with their aid the dentist has the power of doing better work, more conveniently, and in a shorter time, and they will enable him, therefore, to receive a comparatively larger remuneration. We should not ordinarily advise a dentist to try to make use of all these applications at once, but rather to get those that seem most important for his work first, and let the others follow gradually. The old-time advice applies,—slow but sure.

We have not treated this subject at all completely,—indeed, it would take a small volume to do so; but we hope we may have aroused a new and lasting interest in the use of electricity in dentistry. Gentlemen, we thank you for your kind attention.

